# Unprotected water sources and low latrine coverage are contributing factors to persistent hotspots for schistosomiasis in western Kenya

**DOI:** 10.1371/journal.pone.0253115

**Published:** 2021-09-17

**Authors:** Rosemary M. Musuva, Maurice R. Odiere, Pauline N. M. Mwinzi, Isaiah O. Omondi, Fredrick O. Rawago, Sultani H. Matendechero, Nupur Kittur, Carl H. Campbell, Daniel G. Colley

**Affiliations:** 1 Neglected Tropical Diseases Unit, Centre for Global Health, Kenya Medical Research Institute, Kisumu, Kenya; 2 Neglected Tropical Diseases (NTD) Program, Ministry of Health KNH Grounds, Nairobi, Kenya; 3 Schistosomiasis Consortium for Operational Research and Evaluation (SCORE), Center for Tropical and Emerging Global Diseases, University of Georgia, Athens, Georgia, United States of America; 4 Department of Microbiology, University of Georgia, Athens, Georgia, United States of America; Universidade Guarulhos, BRAZIL

## Abstract

**Background:**

Evidence indicates that whereas repeated rounds of mass drug administration (MDA) programs have reduced schistosomiasis prevalence to appreciable levels in some communities referred to here as responding villages (R). However, prevalence has remained high or less than anticipated in other areas referred to here as persistent hotspot villages (PHS). Using a cross-sectional quantitative approach, this study investigated the factors associated with sustained high *Schistosoma mansoni* prevalence in some villages despite repeated high annual treatment coverage in western Kenya.

**Method:**

Water contact sites selected based on observation of points where people consistently go to collect water, wash clothes, bathe, swim or play (young children), wash cars and harvest sand were mapped using hand-held smart phones on the Commcare platform. Quantitative cross-sectional surveys on behavioral characteristics were conducted using interviewer-based semi-structured questionnaires administered to assess water usage/contact patterns and open defecation. Questionnaires were administered to 15 households per village, 50 pupils per school and 1 head teacher per school. One stool and urine sample was collected from 50 school children aged 9–12 year old and 50 adults from both responding (R) and persistent hotspot (PHS) villages. Stool was analyzed by the Kato-Katz method for eggs of *S*. *mansoni* and soil-transmitted helminths. Urine samples were tested using the point-of-care circulating cathodic antigen (POC-CCA) test for detection of *S*. *mansoni* antigen.

**Results:**

There was higher latrine coverage in R (n = 6) relative to PHS villages (n = 6) with only 33% of schools in the PHS villages meeting the WHO threshold for boy: latrine coverage ratio versus 83.3% in R, while no villages met the girl: latrine ratio requirement. A higher proportion of individuals accessed unprotected water sources for both bathing and drinking (68.5% for children and 89% for adults) in PHS relative to R villages. In addition, frequency of accessing water sources was higher in PHS villages, with swimming being the most frequent activity. As expected based upon selection criteria, both prevalence and intensity of *S*. *mansoni* were higher in the PHS relative to R villages (prevalence: 43.7% vs 20.2%; P < 0.001; intensity: 73.8 ± 200.6 vs 22.2 ± 96.0, P < 0.0001), respectively.

**Conclusion:**

Unprotected water sources and low latrine coverage are contributing factors to PHS for schistosomiasis in western Kenya. Efforts to increase provision of potable water and improvement in latrine infrastructure is recommended to augment control efforts in the PHS areas.

## Introduction

Schistosomiasis is a parasitic disease caused by members of the *Schistosoma* species. Globally, approximately 700 million people are at risk of infection [1,2]. More than 240 million people in 78 countries are estimated to be infected with schistosomes. Over 90% of the cases occur in sub-Saharan Africa, where the infection is estimated to cause more than 200,000 deaths annually [3,4].

The current mass drug administration (MDA) strategy recommended by the World Health Organization (WHO) has been shown to be largely effective in reducing schistosomiasis prevalence and intensity at the community level. This strategy involves distribution of praziquantel (PZQ) based on the prevalence of infection in school-aged children (SAC) [5] Evidence indicates that while MDA programs have reduced prevalence to appreciable levels in some areas, prevalence has remained persistently high in other areas, or the reduction in prevalence was less than what was anticipated [6–10] Research findings from our study and others show that transmission is not effectively interrupted by mass drug delivery, especially for high risk locations [11–13]. After more than 20 years of MDA in the Nile Delta, certain villages possessed high *S*. *mansoni* infection prevalence [14]. In western Cote d’Ivoire, an overall reduction in *S*. *mansoni* infection prevalence and intensity was achieved one year after a single, school-based MDA, yet 10% of schools had an increase in *S*. *mansoni* infection prevalence by 25% or more [15]. In a cross-sectional study of 22 villages in the Philippines, 75.6% of participants reported being treated in the last 2 years, yet 13 of those 22 villages had a prevalence above 25% [16].

The sustained prevalence in some of these persistent hotspot (PHS) villages might be attributable to the fact that praziquantel treatment may not be fully curative (juvenile worms are refractory), lack of participation [17], reduced praziquantel effectiveness [18,19], but also on water contact [20]and what influence local environmental and behavioral factors have on individual risk for primary infection and/or reinfection [1,13,21]. In May 2012, through World Health Assembly Resolution 65.21, the World Health Organization called for nations endemic for schistosomiasis to adopt intensive control programs including water, sanitation and hygiene education (WASH) in order to achieve elimination of transmission where feasible [2]. Indeed, a systematic review and meta-analysis suggests that increasing access to safe water and adequate sanitation are important measures to reduce the odds of schistosome infection [22]. The integration of WASH significantly reduced infections with *Ascaris lumbricoides* and *Trichuris trichiura* in addition to *S*. *japonicum* in the People’s Republic of China [23]. *S*. *mansoni* and STH prevalences declined during a trachoma control program in Ethiopia, which increased the use of improved water sources and latrines [24]. In Kenya, a school WASH intervention reduced *A*. *lumbricoides* infection above provision of MDA alone [25].

In order to ensure success, it has been recommended that in large population-based control programs, treatment allocation strategies need to be tailored to local conditions on a village-by-village basis [13]. Factors associated with sustained high prevalences in the PHS villages need to be investigated and addressed if global control efforts and the 2025 elimination targets are to be realized. The objective of this study was to investigate the factors associated with sustained high prevalence for *S*. *mansoni* in some PHS villages in western Kenya despite repeated high annual treatment coverage compared to villages that responded (R) to the annual MDAs.

## Methods

### Study area and population

Villages for inclusion into this study were purposively selected from villages that participated in our previous Schistosomiasis Consortium for Operational Research and Evaluation (SCORE) studies. This preventive chemotherapy-based operational research study was undertaken to control schistosomiasis in this region by implementing school-based and village-wide annual mass drug administration (MDA) in varying treatment regimens for 5 consecutive years (2011–2015) in 150 Kenyan villages situated on or near the eastern shore of Lake Victoria [11]. Six villages with the greatest decrease in *S*. *mansoni* prevalence (Responder villages or R) and another six where prevalence remained persistently high (persistent hotspots or PHS) were selected for inclusion. The specific definition used was the following: a persistent hotspot was defined as a village that had either 25–49% or ≥ 50% prevalence at baseline and having ≥ 10% prevalence in year 5 if starting prevalence was 25–49% or ≥ 25% if starting prevalence was ≥ 50%. Convenience sampling was then used to identify 50 adults from 50 households in each of the 12 villages. Informed consent was obtained from study participants prior to enrolment into the study. A pre-designed and tested questionnaire was administered to the participants and a single stool and urine sample was collected for testing at the KEMRI laboratory.

### Community entry and stakeholder meetings

In order to create Programme ownership and solicit support, stakeholder meetings were held to galvanize interest, support and participation in the study. Before the study commencement, the study team visited the villages and held sensitization meetings with head teachers, community health volunteers and village elders. The health teachers in the study schools were invited to a training workshop and were instructed on the collection of samples, what records and reports were needed for the study, and how to recognize serious adverse events (SAEs) associated with treatment. The phone numbers of the field coordinator and the study nurse were given to the head teacher so that any side effects associated with treatment could be attended to expeditiously.

### Mapping of schools and water contact sites

In all primary schools participating in the study, water contact sites were mapped using hand-held smart phones on the Commcare platform. Water contact sites were selected on the basis of observation of places where people consistently go to collect water, wash clothes, bathe, swim or play (young children) and where there were car washing and sand harvesting activities. Only one GPS point was collected in each school, taking the GPS reading in the center of the school. GPS coordinates on the water bodies were confirmed by following a random sample of 9–12 year olds. The shortest distance from a school or village to a documented transmission site was calculated using QGIS Version 2.18.

### Migration pattern

Data on migration pattern into and out of the villages was collected using key informant interviews with the village elders to understand the level of itinerancy in the villages.

### Water, Sanitation and Hygiene (WASH) surveys

Quantitative cross-sectional surveys on behavioral characteristics using interviewer-based semi-structured questionnaires were administered to assess water usage/contact patterns and open defecation. The level of sanitation at the school, household and community within the villages was determined. The WASH Questionnaire was administered to the head teacher or designated teacher for the WASH survey in schools. Questionnaires were also administered to the household head and pupil. Questionnaires were administered to 15 households per village, 50 pupils per school and 1 head teacher per school.

### Parasitological assessment

Single stool samples from 50 school children aged 9–12 year old and 50 adults in each village were analyzed by the Kato-Katz technique [26] and 4 slides were microscopically examined for eggs of *S*. *mansoni* and soil-transmitted helminths (*Ascaris lumbricoides*, *Trichuris trichiura* and hookworms). Approximately 41.7 mg of faeces were used for each slide. Eggs were counted and expressed in eggs per gram (EPG) by two independent microscopists and any discrepancy in results of the two were reconciled by comparing to results of a third independent and more experienced microscopist. Examination of slides for hookworm eggs was performed within 1 hour of slide preparation. For all other helminths, slides were allowed to clear for at least 24 hours and eggs were counted within one month. Intensity of infection for each helminth was categorized according to the World Health Organization (WHO) proposed thresholds [5].

Individuals who tested positive for schistosomiasis and STH were treated by a qualified study nurse following the recommended dosage by the WHO using a dose pole for praziquantel. These individuals were reached through the help of the community health volunteer in their respective villages.

### Analyses of urine samples

Urine samples (50 from children and 50 from adults) were tested for positivity and band intensity using the point-of-care circulating cathodic antigen (POC-CCA) test (Rapid Medical Diagnostics, Pretoria, South Africa, Batch # 170622073) for detection of *S*. *mansoni* antigen. Briefly, two drops of urine were added to the well of the testing cassette and allowed to absorb. The assay was allowed to develop for 20 min, at which time the results were then read. The test was considered invalid if an internal control band did not appear or if the test was left to develop for more than 25 min after addition of the urine before being read. To score the intensity of the POC-CCA assay results, the intensity of the test band was compared to that of the control band. Positive results were given a score of “Trace” if the band was barely visible, 1+ if the test band was readily visible but less intense than the control band, 2+ if the test band was of equal intensity as the control band, and 3+ if the test band was more intense than the control band. All urine samples were also tested for haematuria using dipsticks to account for the possibility that blood in urine may cause false positives.

### Assessment of fecal occult blood as proxy for intestinal morbidity

Fecal occult blood (FOB) was used as a proxy for intestinal morbidity associated with *S*. *mansoni* in 320 stool samples. FOB was detected using a chromatographic FOB detection test (Mission TestH, Acon Laboratories, San Diego, CA), following the manufacturer’s instructions. Briefly a small amount of feces was homogenised in a liquid buffer provided in the kit after collection. Two drops of stool suspension were applied to a test cassette and results visually read after five minutes and categorized as negative (-) and positive (+).

### Data analyses

Data were entered and transmitted to a central server using phone-based Dimagi Commcare application and data cleaning including consistency and accuracy checks were performed. Data analysis was performed using *STATA* version 14.0. Presence of schistosomiasis, demographic, socioeconomic, environmental and behavioral characteristics in hotspots and responding villages were treated as categorical variables and presented as frequencies and percentages.

Chi-square test for independence was used to examine the significance of the associations and differences in frequency distribution of variables. Odd ratios (OR) and 95% confidence intervals (CI) from univariate logistic regression were used to evaluate the strength of association for variables that were significant in the chi-square test of association. Backward elimination technique was employed to arrive at the final multivariate logistic regression model, beginning with a model containing all the plausible predictors of schistosomiasis and sequentially dropping the variables starting with those that were least significant until the final model where all predictors had a *P*-value < = 0.15. This final model was then used to identify the factors significantly associated with PHS/R villages while adjusting for confounders. All tests were considered significant at *P* < 0.05.

### Ethical considerations

The study was reviewed and approved by KEMRI’s Scientific and Ethics Review Unit (SERU), protocol # 3267. Adults and parents or guardians of children participating in the study provided written informed consent. School children also provided informed assent.

## Results

### Characteristics of study participants

Overall, a majority of the adult population had attained primary education as their highest education level. This trend was similar in the PHS villages and R villages. Most participants in the PHS villages were business owners (29%) followed by farming activities. This was slightly different in the responding villages as farming accounted for 49% while business activities accounted for 25% (**Table 1**). On average, there were 122±54 girls and 131±55 boys in each of the schools. The student population in schools within PHS villages was considerably higher in comparison to those in R villages (**Table 1**).

**Table 1:** General characteristics of the study population.

	Overall, n = 600 (%)	PHS villages n = 300 (%)	R villages n = 300 (%)
**Adult population–n (%)**			
***Level of Education***			
None	56 (9.3)	24 (8.0)	32 (10.7)
Primary not completed	226 (37.7)	98 (32.7)	128 (42.7)
Primary education	171 (28.5)	92 (30.7)	79 (26.3)
Secondary not completed	61 (10.2)	30 (10.0)	31 (10.0)
Secondary education	67 (11.2)	46 (15.3)	21 (7.0)
Post-secondary/college	19 (3.2)	10 (3.3)	9 (3.0)
***Main source of Income***			
Farming	229 (38.2)	83 (27.9)	146 (48.8)
Salaried work	20 (3.3)	13 (4.3)	7 (2.3)
Own Business	162 (27.0)	86 (28.9)	76 (25.3)
Fishing	94 (15.7)	67 (22.3)	27 (9.0)
Skilled labor	79 (13.2)	39 (13.0)	40 (13.3)
**Student population**			
Enrolments in Schools–Mean+ = (Std..Dev)			
Girls	158 +(75.0) =	207 +(60.0) =	109+(54.0) =
Boys	160 +(70.0) =	205+ (51.0) =	116 +(57.0) =

### Parasitological outcomes

As expected, based upon our selection criteria, we confirmed that the prevalence of *S*. *mansoni* by Kato-Katz was more than double (*P* < 0.0001) in the PHS relative to the R villages, whereas the prevalence of soil-transmitted helminths was similar between the PHS and R villages (**Table 2**). There was a higher proportion of moderate and heavy *S*. *mansoni* infections in PHS relative to R villages (**Table 2**).

**Table 2:** Prevalence of *Schistosoma mansoni* and soil-transmitted helminths in PHS and R villages in western Kenya.

	PHS villages n = 600	R villages n = 600	P-value
**By Kato-Katz**	n, (%)	n, (%)	
*Schistosoma mansoni prevalence*	262 (43.7)	121 (20.2)	<0.001*
Light	150 (25.0)	89 (14.3)	
Moderate	84 (14.0)	21 (3.5)	0.005*(by p<0.05)
Heavy	27 (4.5)	10 (1.7)	
Intensity (epg)^1^	73.8 ± 200.6	22.2 ± 96.0	<0.001*
One or more soil-transmitted helminth	25 (4.2)	28 (4.7)	0.673
Hookworm	7 (1.2)	8 (1.3)	0.795
*Ascaris lumbricoides*	5 (0.8)	5 (0.8)	1.000
*Trichuris trichiura*	15 (2.5)	15 (2.5)	1.000
**By POC-CCA**			
*Schistosoma mansoni*	447 (74.5)	283 (47.2)	<0.0001*
Trace	184 (30.7)	140 (23.3)	
*+*	94 (15.7)	68 (11.3)	<0.0001
*++*	109 (18.2)	46 (7.7)	
*+++*	60 (10.0)	29 (4.8)	
**Urine dipstick**	39 (6.5)	36 (6.0)	0.721

Intensity of infection expressed as arithmetic mean ± SD.

*Statistically significant.

^1^Intensity of infection expressed as arithmetic mean ± SD.

**Fig 1:**
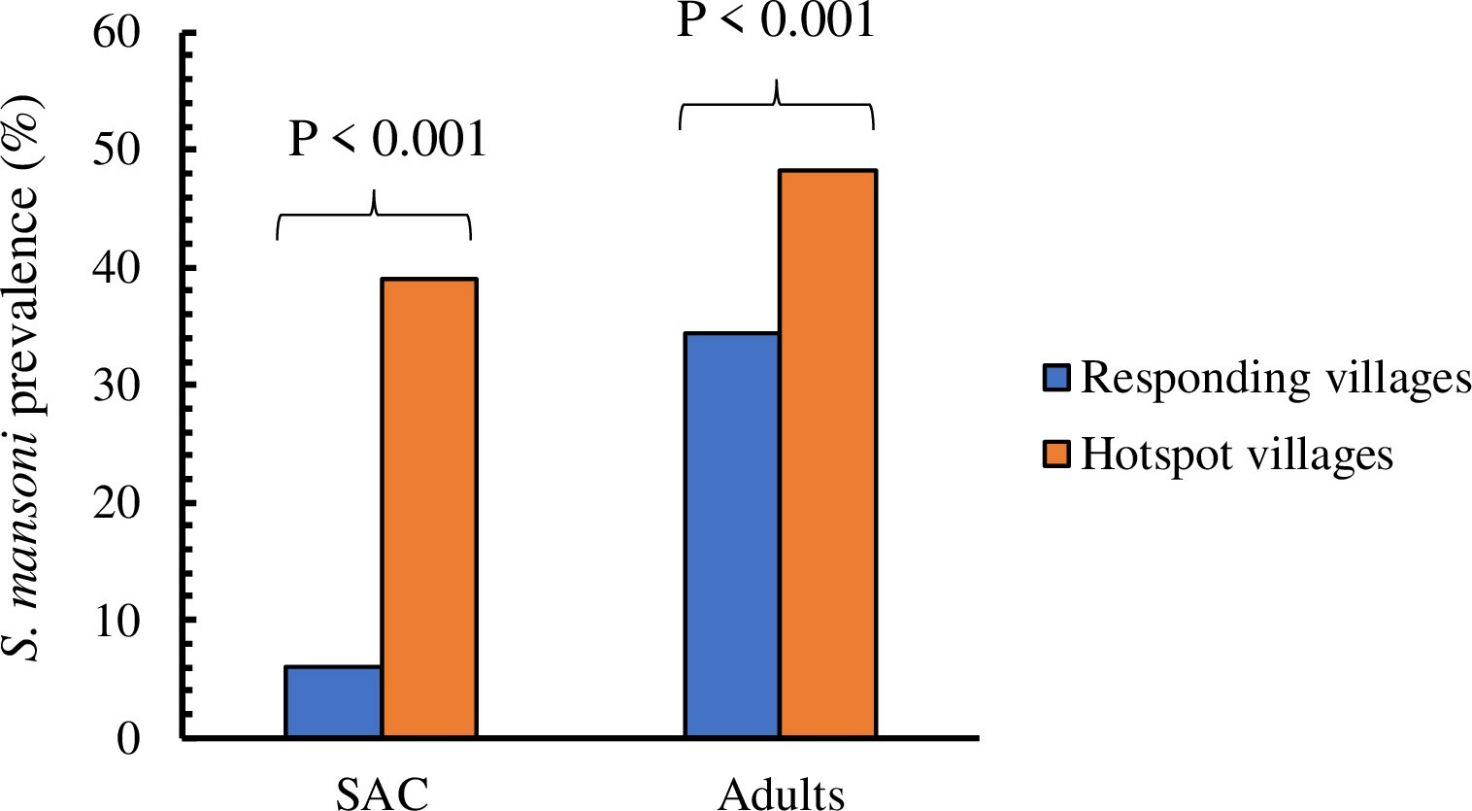
Prevalence of *Schistosoma mansoni* for PHS and R villages by Kato-Katz in 2018.

The trend for higher *S*. *mansoni* prevalence in PHS was also evident when the data was analyzed by age group (**Fig 1**). For the SAC population, all PHS villages had higher *S*. *mansoni* prevalence compared to R villages, whereas for the adult population, this held true except for one village (Mumbo) in the R arm located in Siaya County which had high *S*. *mansoni* prevalence of 54%.

**Fig 2:**
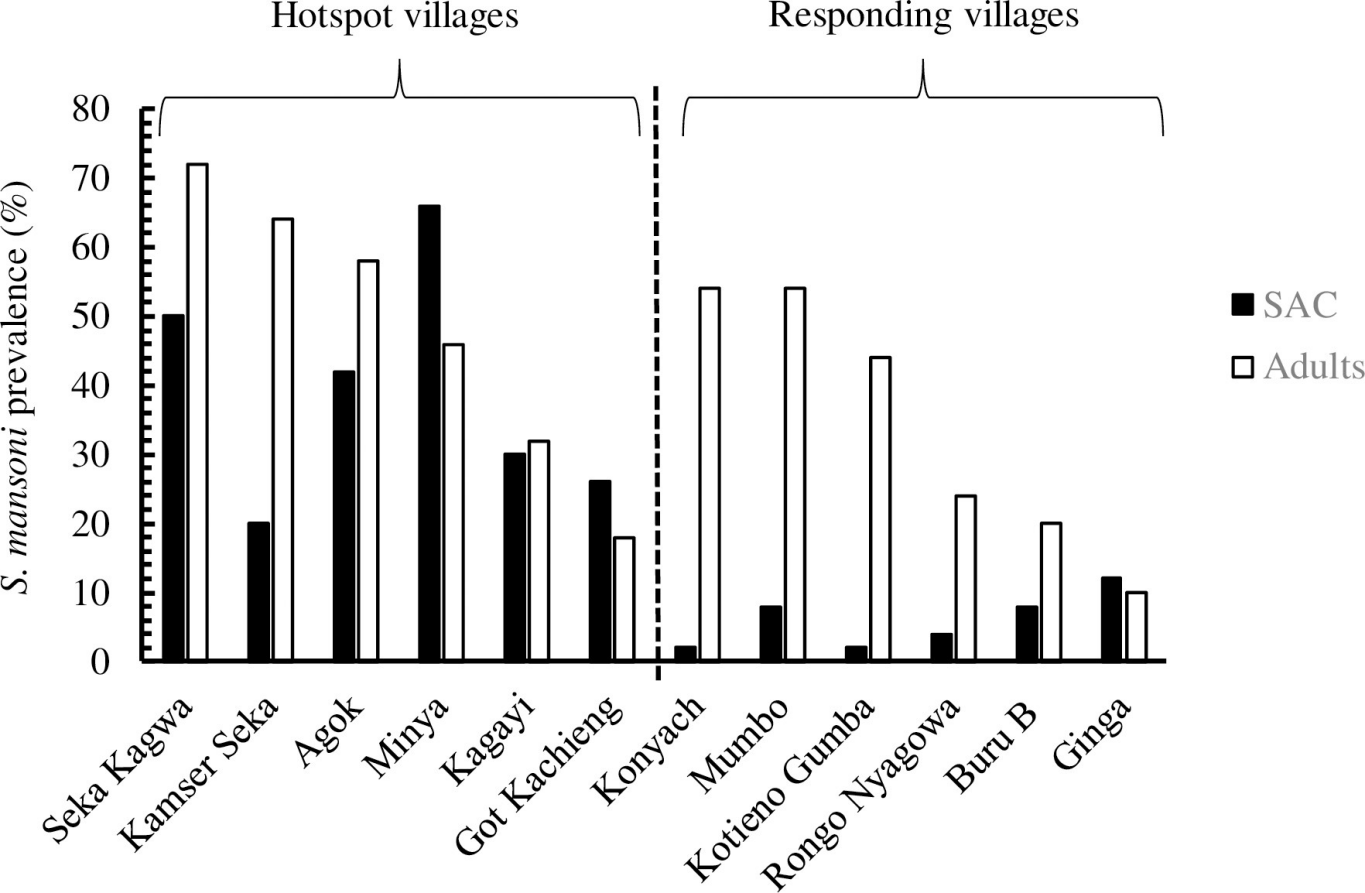
The prevalence of *Schistosoma mansoni* in PHS and R villages by age group (SAC age; adults age, in 2018).

In the PHS villages, Seka Kagwa had the highest *S*. *mansoni* prevalence (72.0%) and Got Kachieng’ the lowest prevalence (18%) among adults, whereas Minya village had the highest prevalence (66%) and Kamser Seka the lowest prevalence (20%) among SAC (**Fig 2**). In the R villages, Mumbo and Konyach had the highest prevalence (54% each) and Ginga the lowest prevalence (10%) among adults, whereas Ginga had the highest prevalence (12%) and Kotieno Gumba and Konyach the lowest prevalence (2% each) among SAC (**Fig 2**).

A similar trend of higher *S*. *mansoni* prevalence in PHS (74.5%) relative to R villages (47.2%) was observed when comparisons were made based on POC-CCA analyses. The prevalence of *S*. *mansoni* by POC-CCA was almost double (P < 0.0001) in the PHS relative to the R villages (**Table 2**). In general, there was a higher proportion of 2+ and 3+ intensity of infection as depicted by POC-CCA in the PHS relative to R villages (**Table 2**). All traces were considered positive following the manufacturer’s recommendations.

#### Urine dipstick results

There was no significant difference in microhematuria results between PHS and R villages (**Table 2**).

### Intestinal morbidity

Of the 320 stool samples tested for intestinal morbidity using FOB, 35 (10.9%) were positive for traces of blood.

### Migration into and out of villages

Movement into the villages was reported to be majorly driven by festivals (27.5%) and other reasons such as gatherings by local administration (Chief’s *barazas*), fishing, funerals and sports (32.5%). In the PHS such movements into PHS villages were majorly attributed to attendance of Chief’s *barazas*, fishing, funeral and sports (37.5%) while in the R villages they were due to attendance of markets (31.3%) and festivals (25.0%). However, there was no significant difference in the movement into PHS and R villages (*χ*^2^ = 1.8888, *P* = 0.756). On average, about 75 people and about 50 people come to PHS and R villages, respectively, for various reasons, although there was no significant difference in the number of people moving into PHS and R villages (Z = -0.236; *P* = 0.8131) (**Table 3**).

**Table 3:** Migration into and out of the PHS and R villages.

	Overall	PHS villages	R villages	P-value
MOVEMENT INTO THE VILLAGE–n (%)				
Reasons for movement into the village				
Market	10 (25.0)	5 (20.8)	5 (31.3)	0.756
Festival	11 (27.5)	7 (29.2)	4 (25.0)	
Permanent relocation	6 (15.0)	3 (12.5)	3 (18.8)	
Other reasons e.g. *Barazas*, fishing, funerals, sports	13 (32.5)	9 (37.5)	4 (25.0)	
Approximate number of people moving into village–Median (Range)	50 (20–200)	50 (25–200)	75 (10–200)	0.8131
MOVEMENT FROM THE VILLAGE				
Reasons for movement from the village				
Market	11 (27.5)	7 (29.2)	4 (28.6)	0.801
Festival	13 (32.5)	7 (29.2)	6 (42.9)	
Permanent relocation	6 (15.0)	4 (16.7)	2 (14.3)	
Other reasons e.g. *Barazas*, fishing, funerals, sports	8 (5.0)	3 (30.0)	1 (11.1)	
Approximate number of people moving out of village—Median(Range)	50 (20–100)	40 (14–110)	75 (30–100)	0.4020

Movement from all the villages as a whole was primarily related to attendance at festivals (32.5%) and markets (27.5%). There were some differences when analyzing the villages by PHS and R. In the PHS villages such movements were mainly related to attendance of markets (29.2%) and festivals (29.17%) while in the R villages they were mainly driven by attendance of festivals (42.7%). There was no significant difference in the reasons for movement out of the villages when PHS and responding villages were compared (*χ*^2^ = 0.9994, *P* = 0.801).

On average, 50 people per month come to and from these villages for the various reasons noted above with about 40 people in PHS and 75 in R villages. Although there were more people moving into and out of R villages compared to PHS villages, there was no significant difference from PHS and R villages (Z = 0.838, *P* = 0.4020) (**Table 3**).

### Water, sanitation and hygiene at the household level

#### Sources of water

Overall, 77.3% of the study population had latrines in their compounds. Moreover, latrine coverage was higher within R villages compared to PHS villages (83.7% versus 71.0%) (**Table 4**). With regard to the source of water for domestic use, a majority of the study population reported to be using surface water and unprotected springs (60.5% and 11.3%, respectively) for bathing and (47.8% and 10.3%, respectively) for drinking; 81.5% reported to visit surface water sources at least daily (**Table 4**).

**Table 4:** Descriptive statistics on water, sanitation and hygiene in study villages based on village data from household heads.

	Overall (n = 600)	PHS villages (n = 300)	R villages (n = 300)
Latrine present	464 (77.3)	213 (71.0)	251 (83.0)
**Source of bathing water**			
Unprotected spring	68 (11.3)	50 (16.7)	18 (6.0)
Protected spring	0	0	0
Unprotected dug well	53 (8.8)	42 (14.0)	11 (3.7)
Protected dug well	18 (3.0)	1 (0.3)	17 (5.7)
Hand pump/tube well/borehole	38 (6.3)	9 (3.0)	29 (9.7)
Surface water (river/ stream, dam, lake, canal)	363 (60.5)	194 (64.7)	169 (56.3)
Public piped water/tap/standpipe	38 (6.3)	1 (0.3)	37 (12.3)
Rain water collection	9 (1.5)	3 (1.0)	6 (2.0)
In home piped water/tap/standpipe	13 (2.2)	0	13 (4.3)
Mobile water tanker	0	0	0
**Source of drinking water**			
Unprotected spring	62 (10.3)	47 (15.7)	15 (5.0)
Protected spring	5 (0.8)	1 (0.3)	4 (1.3)
Unprotected dug well	26 (4.3)	24 (8.0)	2 (0.7)
Protected dug well	14 (2.3)	0	14 (4.7)
Hand pump/tube well/borehole	54 (9.0)	15 (5.0)	39 (13.0)
Surface water (river/ stream, dam, lake, canal)	287 (47.8)	173 (57.7)	114 (38.0)
Public piped water/tap/standpipe	91 (15.2)	22 (7.3)	69 (23.0)
Rain water collection	48 (8.0)	17 (5.7)	31 (10.3)
In home piped water/tap/standpipe	13 (2.2)	1 (0.3)	12 (4.0)
Mobile water tanker	0	0	0
Visit at least one surface water source	487 (81.5)	260 (86.7)	227 (75.7)
Weekly Number of times of contacting surface water Mean(Std.dev)	9.6 (9.5)	11.2 (10.9)	7.9 (7.3)
Number of times accompanied by child	2.2 (3.6)	2.6 (3.8)	1.7 (3.3)

Results for univariate logistic regression for association between water, sanitation and hygiene between PHS and R villages for data from household heads is presented in **Table 5**. Latrine coverage was more widespread in R villages than PHS villages. R villages were associated with more than 2 times higher odds of having latrines (OR = 2.092; 95% CI 1.410–3.105; *P* < 0.0001). In addition, a higher percentage of the residents of R villages used protected water sources for both bathing (34.0% vs 4.7%) and drinking (56.3% vs 18.7%). R villages were associated with higher odds of using protected water sources for bathing (OR = 10.5; 95% CI 5.850–18.93; *P* < 0.0001) and drinking (OR = 5.62; 95% CI 3.885–8.132; *P* < 0.0001). 87.3% of the residents from PHS villages visited surface water sources at least on a daily basis as compared to their counterparts from R villages where only 75.7% visited the sites. R villages therefore were associated with lower odds of visiting such sites (OR = 0.454; 95% CI 0.295–0.699; *P* < 0.0001) (**Table 5**).

**Table 5:** Univariate logistic regression on the nature of water, sanitation and hygiene in study villages from household heads.

	Overall (n = 600)	PHS villages (n = 300)	R villages (n = 300)	OR (95% C.I)	P-value
Latrine present	464 (77.3)	213 (71.0)	251 (83.7)	2.09 (1.410–3.105)	<0.0001
*Source of bathing water*					
Protected water source	116 (19.3)	14 (4.7)	102 (34.0)	10.52 (5.85–18.93)	<0.0001
*Source of drinking water*					
Protected water source	225 (37.5)	56 (18.7)	169 (56.3)	5.62 (3.885–8.132)	<0.0001
Visit at least one surface water source	487 (81.2)	260 (87.3)	227 (75.7)	0.45 (0.295–0.699)	<0.0001

All variables that were non-significant in the univariate analysis were removed from the model building process, which then proceeded using a forward model building technique. Starting with a model containing only an intercept, variables were added to the model one at a time based upon significant contribution relative to the contribution of the other variables. The analysis included 600 adults; Odds ratios adjusted for all variables are included in the Table. The results indicated that the factors associated with the PHS villages were: absence of latrines (OR = 2.25; 95% CI = (1.43–3.53); *P* = 0.000); use of unprotected bathing water sources (OR = 5.05; 95% CI = (2.38–10.73); *P* = 0.000); use of unprotected drinking water sources (OR = 3.58; 95% CI = (2.24–5.74); *P* = 0.000) and poverty (OR = 2.34; 95% CI = (1.58–3.47); *P* = 0.000) (**Table 6**).

**Table 6:** Multivariate analysis of the relation between PHS villages and other indicators in the adult population.

Characteristic	Adj.OR	95% CI	P-value
Presence of latrine			
Yes	1.0	-	
No	2.25	1.43–3.53	<0.001
Protected bathing water source			
Yes	1.0	-	
No	5.05	2.38–10.73	<0.001
Protected drinking water source			
Yes	1.0	-	
No	3.58	2.24–5.74	<0.001
Visit surface water sources			
No	1.0	-	
Yes	1.66	0.92–3.00	0.095
Socio-Economic Status			
Less Poor	1.0	-	
Poorest	2.34	1.58–3.47	<0.001

### Knowledge and practice on schistosomiasis among adult population

57.0% of the adult population had ever heard of schistosomiasis, however a marginally higher proportion of those from R villages had ever heard of the disease (58.0%) compared to their counterparts from PHS villages (56.0%); this difference was however not statistically significant (*P* = 0.621) (**Table 7**).

**Table 7:** Knowledge and practice on schistosomiasis among adult population.

	Overall (n = 600)	PHS Villages (n = 300)	R Villages (n = 300)	*P*-value
Ever heard of Bilharzia	342 (57.0)	168 (56.0)	174 (58.0)	0.621
Knowledge on ways of infection	(n = 342)	(n = 168)	(n = 174)	
Correct ways	186 (31.0)	91 (54.2)	95 (54.6)	0.470
Incorrect ways	105 (54.4)	56 (33.3)	49 (28.2)	
Don’t know	51 (14.9)	21 (12.5)	30 (17.2)	
Right place to seek treatment	(n = 342)	(n = 168)	(n = 174)	
Health facility	340 (99.2)	168 (100)	172 (98.9)	0.324
Drug shop	2 (0.6)	0	2 (1.15)	
Traditional healer	0	0	0	

With regards to knowledge about ways in which humans get infected with schistosomiasis, overall 31.0% cited correct ways and this translated to 54.2% of those in PHS villages and 54.6% of those in R villages); this difference was however also not statistically significant (P = 0.470). Of all the 342 adults who reported having ever heard of schistosomiasis, 99.4% regarded health facilities as the right place to seek treatment in case of an infection, while only 0.6% regarded drug shops as a suitable place to obtain schistosomiasis medication.

### Water, sanitation and hygiene at the school level

#### Sources of water

The main sources of drinking water in schools were rain water (83.3%) and surface water (50%). Rainwater was also the mostly used water source for drinking among the students (50%), the average distance from the school to the main water source was 170±177.12 meters (**Table 8**).

**Table 8:** Descriptive statistics on water, sanitation and hygiene in study villages based on data from school heads.

	Overall (n = 600)	PHS villages (n = 300)	R villages (n = 300)	*P*-value
**Source of drinking water in the school–n (%)**				
Unprotected dug well	50 (8.3)	50 (16.7)	0	<0.0001
Surface water	400 (66.7)	250 (83.3)	150 (50.0)	<0.0001
Public piped water	50 (8.33)	0	50 (16.7)	<0.0001
Rain water collection	450 (75.0)	300 (100.0)	150 (50.0)	<0.0001
In school piped water	50 (8.3)	0	50 (16.7)	<0.0001
**Mostly used drinking water source–n (%)**				
None	50 (8.3)	50 (16.7)	0	<0.0001
Unprotected dug well	100 (16.7)	50 (16.7)	50 (16.7)	
Public piped water	50 (8.3)	0	50 (16.7)	
Surface water	150 (25.0)	100 (33.3)	50 (16.7)	
Rain water collection	200 (33.3)	100 (33.3)	100 (33.3)	
In school piped water	50 (8.3)	0	50 (16.7)	
Distance from main water source—Mean (Std.Dev) (metres)	369.1(807.2)	669.2 (1057.9)	69 (73.5)	<0.0001
Latrine type				
Pit latrine without slab or pit	300 (50.0)	150 (50.0)	150 (50.0)	1.0000
Ventilated improved pit latrine (VIP)	300 (50.0)	150 (50.0)	150 (50.0)	
Number of holes–Mean (Std.Dev)	9 (3.0)	9 (3)	10 (3)	0.0348
Number of functional girls’ latrine–Mean (Std.Dev)	5 (1)	5 (1)	5 (1)	1.0000
Number of functional boys’ latrine–Mean (Std.Dev)	5 (2)	4 (2)	4 (2)	1.0000
Presence of latrines for young children–n (%)	200 (33.3)	150 (50.0)	50 (16.7)	<0.0001
Presence of traces of open defecation–n (%)	200 (33.3)	50 (16.7)	150 (50.0)	<0.0001

The main source of drinking water was rain water (100%) and surface water (66.7%) for PHS and rainwater (66.7%) for R schools (**Table 8**).

In schools in the PHS villages, unprotected dug well (33.3%) and surface water (33.3%) were mostly used while in the schools in the R villages rain water was mainly used (66.7%). In addition, schools in PHS villages were further away from main water source (270 ± 201.84 meters) as compared to those in R villages (70 ± 45.61 meters), (*P*<0.0001).

50% of the pupils use protected water sources while at school and a majority of these were from schools within R villages (66.7%) as compared to those from PHS villages (33.3%). Thus, those from schools in R villages were associated with higher odds of using protected water sources as compared to those from PHS schools (OR = 5.0; 95% CI 3.4,7.3; *P* < 0.001) (**Table 9**).

**Table 9:** Univariate logistic regression on the nature of water, sanitation and hygiene in study schools from school children.

	Overall (n = 600)	PHS villages (n = 300)	R villages (n = 300)	OR (95% C.I)	*P*-value
*Main source of drinking water at school–n (%)*					
Protected water source	200 (33.3)	50 (16.7)	150 (50.0)	5.0 (3.4–7.3)	<0.001
*Latrine usage–n (%)*					
Urinate in latrine while at school	594 (99.0)	298 (99.3)	296 (98.7)	0.5 (0.1–2.7)	0.421
Defecate in latrine while at school	595 (99.2)	297 (99.0)	298 (99.3)	1.5 (0.2–9.1)	0.656
Always use latrine while at school	356 (59.3)	105 (35.0)	251 (83.7)	9.5 (6.5–14.0)	<0.001
Urinate in latrine while at home	381 (63.5)	181 (60.3)	200 (66.7)	1.3 (0.9–1.8)	0.107
Defecate in latrine while at home	523 (87.2)	248 (82.7)	275 (91.7)	2.3 (1.4–3.8)	0.001
*Meet WHO latrine-pupil ratio–n (%)*					
For boys (1:50)	350 (58.3)	100 (33.3)	250 (83.3)	10.0 (6.8–14.7)	<0.001
For girls (1:25)	150 (25.00)	0	150 (50.00)	-	<0.001
Share latrine with other members of the household	265 (47.5)	146 (48.7)	119(39.7)	0.6 (0.4–0.8)	0.001

All variables at school level that were non-significant in the univariate analysis were removed from the model building process, which then proceeded using a forward model building technique. Starting with a model containing simply an intercept, variables were added to the model one at a time based upon significant contribution relative to the contribution of the other variables. The analysis included 600 SAC; Odds ratio adjusted for all variables included into the Table.

The results indicated that the schools within PHS villages were associated with increased odds of: using water from unprotected water sources for drinking (OR = 4.38; 95% CI = (1.98–9.72); *P* = 0.000); decreased odds of defecating in latrine while at home (OR = 0.35; 95% CI = (0.12–1.00); *P* = 0.050); inconsistent use of school latrines (OR = 10.49; 95% CI = (5.21,21.14); *P* = 0.000); inability to meet WHO latrine-pupil ratio (OR = 6.56; 95% CI = (2.94–14.64); *P* = 0.000); sharing latrines with other members of the household (OR = 2.16; 95% CI = (1.15–4.06); p = 0.016); decreased odds of bathing in unsafe water bodies (OR = 0.4; 95% CI = (0.19,0.86); *P* = 0.019); playing in unsafe water bodies (OR = 8.30; 95% CI = (2.81,24.81); *P* = 0.000); swimming in unsafe water bodies (OR = 8.76; 95% CI = (4.35–17.67); *P* = 0.000); visiting surface water sources on a daily basis, (OR = 7.94; 95% CI = (2.92–21.64); *P* = 0.000) (**Table 10**).

**Table 10:** Multivariate analysis of the relation between PHS villages and other indicators in the school children population.

Characteristic	Adj.OR	CI (95%)	*P*-value
*Main source of drinking water at school*			
Protected	1.00	-	
Unprotected	4.38	1.98–9.72	<0.0001
Defecate in latrine while at home			
Yes	1.00	-	
No	0.35	0.12–1.00	0.050
Consistent use of school latrines			
Yes	1.00	-	
No	10.49	5.21–21.14	<0.0001
*Meet WHO latrine-pupil ratio (for boys)*			
Yes	1.00	-	
No	6.56	2.94–14.64	<0.0001
Share latrine with other members of the household			
No	1.00	-	
Yes	2.16	1.15–4.06	0.016
Number of days missed in the past 2 weeks			
Zero days	1.00	-	
1–2 days	0.60	0.28–1.25	0.168
3–5 days	0.92	0.20–4.23	0.916
>5 days	4.24	0.90–19.97	0.068
Bathe in water bodies			
No	1.00	-	
Yes	0.4	0.19–0.86	0.019
Wash clothes in water bodies			
No	1.00		
Yes	1.55	0.76–3.16	0.231
Fetch water from water bodies			
No	1.00	-	
Yes	1.44	0.65–3.18	0.366
Play in water bodies			
No	1.00	-	
Yes	8.3	2.81–24.51	<0.0001
Swim in water bodies			
No	1.00	-	
Yes	8.76	4.35–17.67	<0.0001
Weekly Frequency of visiting water contact sites			
Once/twice a week	1.00	-	
More than twice	1.30	0.66–2.54	0.445
Daily	7.94	2.92–21.64	<0.0001

#### Water contact pattern

84.2% of the students had at least one surface water body close to their home/school. This translated to 89.3% of those in schools within PHS villages and 79.0% of those in schools within R villages.

Most students visited the surface water bodies to bathe (68.8%), to wash clothes (64.5%) and to fetch water (64.7%); majority of those in PHS schools visited such water bodies to bathe (76.0%), to wash clothes (72.3%) and to fetch water (74.0%); whereas those in R villages also went to such water sources to bathe (61.7), to wash clothes (56.7%) and to fetch water (55.3%) (**Table 11**).

**Table 11:** Water contact patterns in schools.

	Overall (n = 600)	PHS Villages (n = 300)	R Villages (n = 300)	*P*-value
Have water body near school/home	505 (84.2)	268 (89.3)	237 (79.0)	0.001
*Activities on water body*				
Bathing	413 (68.8)	228 (76.0)	185 (61.7)	<0.0001
Washing clothes/dishes	387 (64.5)	217 (72.3)	170 (56.7)	<0.0001
Fishing	18 (3.0)	7 (2.3)	11 (3.7)	0.338
Crossing	4 (0.7)	3 (1.0)	1 (0.3)	0.316
Fetching water	388 (64.7)	222 (74.0)	166 (55.3)	<0.0001
Playing	118 (19.7)	109 (36.3)	9 (3.0)	<0.0001
Swimming	188 (31.3)	151 (50.3)	37 (12.3)	<0.0001
*Frequency of visiting water contact sites*				
Daily	264 (44.0)	156 (58.7)	108 (47.0)	0.002
Once/Twice a week	178 (29.7)	77 (28.9)	101 (43.9)	
More than twice per week	54 (9.0)	33 (12.4)	21 (9.1)	

With regards to frequency of visiting environmental water sources, 44.0%, 29.7% and 9.0% visited such sources daily, weekly and more than twice a week, respectively. A higher proportion of those from PHS villages visited such water bodies daily as compared to those from R villages (58.7% vs. 47.0%, *P* = 0.002) (**Table 11**).

#### Latrine coverage in schools and homes from the pupil questionnaire

The available latrine types were pit latrine without slab/pit (50%) and Ventilated Improved Pit (VIP) latrine (50%); the average number of holes in the latrines were 9±3; the number of functional latrines were 5±1 and 4±2 for the girls and boys respectively; Only 16.67% of the schools had latrines for young children whereas there were traces of open defecation in 33.33% of the schools (**Table 8**).

Schools within PHS villages mainly had Ventilated Improved Pit latrine (66.7%) while those in R villages mostly had pit latrine without slab/pit (66.67%). Surprisingly, schools within PHS villages had more number of holes than those in R villages (9±3 vs 8±2, P = 0.0009); latrines for young children were only present in PHS (33.33%) schools and open defecation traces were only found in R villages (66.67%) (**Table 8**).

Out of the 600 students interviewed, 99% reported to be urinating in the school latrines while at school (99.3% and 98.7% in the PHS and R schools respectively); 99.3% reported to be defecating in the school latrine while at school (100% and 98.7% in schools in the PHS and R villages respectively). 53% reported to be consistently using the school latrines while at school (29.3% and 76.7% in schools in the PHS and R villages respectively); 56.3% reported to be urinating in the home latrines while at home (52.0% and 60.7% in schools in the PHS and R villages respectively). 84% reported to be defecating in the home latrines while at home (74.7% and 93.3% in schools in the PHS and R villages respectively). The students from schools within R villages were associated with increased odds of consistently using latrines while at school and of defecating in home latrines while at home, (OR = 9.5; 95% CI, 6.5–14.0; *P*<0.001 and OR = 2.3; 95% CI, 1.4–3.8; *P* = 0.001 respectively) (**Table 9**).

66.7% of the schools met the WHO recommended latrine-pupil ratio for boys (33.3% and 100% of the schools within PHS and R villages respectively) while 33.3% of the schools met the WHO recommended latrine-pupil ratio for girls (0% and 66.67% of the schools within PHS and R villages, respectively). Schools within R villages were 10 times more likely to meet WHO pupil-latrine standard as compared to those in PHS villages (OR = 10.0; 95% CI, 6.0–14.7; *P*<0.001). Therefore, schools within PHS villages had poor latrine to pupil ratio as compared to schools in R villages with regards to the recommended latrine-pupil ratio standard (**Table 9**).

47.5% of the students who had latrines at home, shared the latrines with other members of their respective household, this corresponded to 48.7% of those in PHS villages and 39.7% of those in R villages. Moreover, R villages were associated with reduced odds of latrine sharing than PHS villages (OR = 0.6; 95% CI = 0.4–0.8; *P* = 0.001).

## Discussion

In this cross-sectional, school-based and community-wide study of children and adults in PHS and responding villages, where mass chemotherapy has been implemented for several years, we found higher prevalence of *S*. *mansoni* in the PHS relative to the R villages. Our data suggests that this sustained higher *S*. *mansoni* prevalence in PHS villages is likely driven by, among other things, frequency of use of unprotected water sources and low latrine coverage. We observed a lower latrine coverage ratio in the PHS relative to R villages, and surprisingly, only 33% of schools in the PHS villages met the WHO threshold for boy: latrine coverage, while none met the girl: latrine ratio requirement.

The prevalence and intensity of *S*. *mansoni* remained high in PHS compared to R villages, a result that is consistent with our previous work [11].

Evidence suggests that PHS villages may take longer to realize reductions in *S*. *mansoni* infections and infection intensity. To address this, more frequent chemoprophylaxis or additional interventions tailored to local settings need to be implemented in such areas. A possible explanation for the hotspot’s existence and persistence includes the presence of super-spreaders, differences in snail populations (such as numbers of snails or differences in snail species) [11]. The shoreline adjacent to the PHS villages is along the main body of Lake Victoria, whereas the responding villages are distributed around the Winam Gulf. The differences in the ecology of these two areas could influence the numbers or relative distribution of appropriate intermediate host snail species, which could result in differences in the force of transmission between the two areas One additional factor could be the role of persistent non-compliant individuals who routinely miss out on MDA for whatever reasons. Our ongoing studies are designed to explore these possibilities.

In our study, a higher proportion of individuals accessed unprotected water sources for both bathing and drinking in PHS relative to responding villages. Frequency of accessing water sources was also higher in PHS, with swimming and playing being most pronounced activities. Consistent with our findings, Nyati-Jokomo and Chimbari [27] found that frequent contact with unprotected water sources for bathing and swimming were risk factors for schistosomiasis among school children in Zimbabwe. Similarly, a separate study in Um-Asher area, Khartoum, Sudan reported that source of drinking water was relatively associated with schistosomiasis [28]. Water contact patterns can vary considerably between households [29], let alone entire villages. In general, most water contact takes place at the nearest sites to the homes with frequency and duration of contact declining slowly with distance. Our findings would indicate the need for provision of potable water and piped water as key interventions to reduce transmission in the PHS. However, provision of safe water in itself may not always produce expected results. For instance, in some settings, other activities such as fishing, sand harvesting and car washing account for considerable occupational water contact that safe water supplies would not prevent transmission [30–32]. Furthermore, the proportion of water contact that continues with safe water supplies may vary widely between different groups of people and between settings, as a result of cultural, environmental and socioeconomic differences [33]. Fishing and sand harvesting are among the key occupational activities for communities around Lake Victoria, and male adults from such PHS would be expected to receive the least benefit from provision of safe water.

Our results on higher frequency of water contacts and associated higher *S*. *mansoni* prevalence are supported with evidence from other settings. Lima e Costa *et al* [34] found that individuals reporting water contact less than once a week had a smaller excess risk of schistosomiasis than those reporting water contact at least weekly (OR 3.0, CI 1.3–6.6 in comparison to OR 4.3, CI 2.6–7.0). In China, Zhaowu *et al*. [35] found that reinfection was associated with the frequency of water contact, the type of water contact and the proximity of residence to snail-infested water. Control interventions that center on water and sanitation provision designed to either decrease contamination of water or decrease human contact with contaminated water would be effective in reducing schistosomiasis in such hotspots.

We observed a higher latrine coverage in the R relative to PHS villages with only 33% of schools in the PHS villages meeting the WHO threshold for boy: latrine coverage while none in the girl: latrine ratio was met. Interestingly, presence of a VIP latrine with a slab in schools within PHS villages seemed not to have a positive influence on reduction of prevalence in these villages. Studies have shown that a pit latrine in itself is not sufficient and there are other socio-cultural practices that may affect its use and consequently its effectiveness. Nevertheless, with regards to overall latrine coverage, R villages were associated with more than 2 times higher odds of having latrines (OR = 2.092; 95% CI 1.410–3.105) and this may be the key driving force. Counter-intuitively, traces of open defecation were observed only in schools in R and not PHS villages. Whereas open defecation is known to increase the risk of infection [36], this phenomenon may simply be related to poor health education among children in schools in R villages and does not seem to play a significant role on transmission given the low *S*. *mansoni* prevalence in R villages.

In general, our findings on WASH are consistent with those of Grimes and others (2014) that found that people with safe water had significantly lower odds of a *Schistosoma* infection, as did those with adequate sanitation. Properly constructed latrines are necessary in order to promote hygiene in schools and among the community [37]. However, while faecal contamination of the area can be reduced by the provision of adequate latrines, it is difficult to prevent children and others from urinating in the water, indicating the need for health education. In addition, construction of such latrines may be hampered by the high water table in such areas. Such high water tables, coupled with black cotton soils and rock outcrops in these areas may also hamper latrine construction and contribute to the low latrine coverage observed in hotspot villages. Whereas the distance between hotspot and responding villages might not be very significant, there are certainly microgeographic variations in topography across villages.

Our suggestion that sustained higher *S*. *mansoni* prevalence in PHS villages is driven by unprotected water sources and poor sanitation is supported by the unique dichotomy that emerges when one considers the prevalence of infection for schistosomiasis and STH between PHS and responding villages. While the *S*. *mansoni* prevalence was higher in the PHS, STH prevalence was similar between the PHS and R villages, suggesting that access to unprotected water sources, probably transmission sites where snail vectors were present explains this phenomenon and not just contaminated environments. Indeed, the impact of sanitation upon schistosome transmission is dependent upon its ability to reduce fecal or urinary contamination of freshwater containing intermediate host snails, rather than contamination of the environment in general. However, we cannot rule out the possible contribution of albendazole administered through the annual Kenya National School-based Deworming Program (NSBDP) on lowering the STH prevalence. Whereas the NSBDP provides both praziquantel and albendazole to schools in endemic areas, MDA by albendazole covers a higher number of schools compared to schools that receive both praziquantel and albendazole.

## Conclusion

Unprotected water sources and low latrine coverage emerged as main contributing factors to PHS villages for schistosomiasis in western Kenya. Efforts to increase provision of potable water and improvement in latrine infrastructure are recommended to augment control efforts in these persistent hotspot areas. Our findings add to the body of evidence [33,38,39] (Grimes *et al* 2015; Utzinger *et al*. 2009; Freeman *et al*. 2013) emphasizing the need for multisectoral and integrated approaches to the control of schistosomiasis and other neglected tropical diseases (NTDs) especially in such persistent hotspot areas.

## Supporting information

S1 Dataset Score dataset. (XLS)

S1 File Data collection tools-completed. (PDF)

## References

[1] Colley DG, Bustinduy AL, Secor WE, King CH, 2014. Human schistosomiasis. Lancet 383: 2253–2264. doi: 10.1016/S0140-6736(13)61949-2 24698483 PMC4672382

[2] WHO, 2012. Preventive Chemotherapy Databank. Geneva, Switzerland: Word Health Organization.

[3] Van der Werf MJ, de Vlas SJ, Brooker S, Looman CW, Nagelkerke NJ, Habbema JD, et al, 2003. Quantification of clinical morbidity associated with schistosome infection in sub-Saharan Africa. Acta Trop 86: 125–139. doi: 10.1016/s0001-706x(03)00029-9 12745133

[4] WHO, 2013. Schistosomiasis: number of people treated in 2011. Wkly Epidemiol Rec 88: 81–88. 23540050

[5] WHO 2002. Prevention and control of schistosomiasis and soil-transmitted helminthiasis: Report of a WHO expert committee. WHO Technical Report Series No. 912. In. Geneva: World Health Organization; 1–57. 12592987

[6] Ahmed AM, El Tash LA, Mohamed EY, Adam I. 2012. High levels of *Schistosoma mansoni* infections among schoolchildren in central Sudan one year after treatment with praziquantel. J Helminthol, 86(2):228–32. doi: 10.1017/S0022149X11000290 21729382

[7] Clements AC, Bosque-Oliva E, Sacko M, Landoure A, Dembele R, Traore M, et al. A comparative study of the spatial distribution of schistosomiasis in Mali in 1984–1989 and 2004–2006. PLoS Negl Trop Dis. 2009;3:e431. doi: 10.1371/journal.pntd.0000431 19415108 PMC2671597

[8] Landoure A, Dembele R, Goita S, et al. Significantly Reduced Intensity of Infection but Persistent Prevalence of Schistosomiasis in a Highly Endemic Region in Mali after Repeated Treatment. PLoS Negl Trop Dis. 2012 Jul;6(7). 10.1371/journal.pntd.0001774 PMC3409132 22860153

[9] Poggensee G, Krantz I, Per Nordin, Sabina Mtweved S, Ahlberg B, Mosha G, et al. 2005. A six-year follow-up of school-children for urinary and intestinal schistosomiasis and soil-transmitted helminthiases in Northern Tanzania. Acta Trop. 9:131–140. 10.1016/j.actatropica.2004.10.003 15652327

[10] Kittur N, Binder S, Campbell CH, King CH, Kinung’hi S, Olsen A, et al (2017) Defining Persistent Hotspots: Areas That Fail to Decrease Meaningfully in Prevalence after Multiple Years of Mass Drug Administration w ith Praziquantel for Control of Schistosomiasis. Am J Trop Med Hyg 97: 1810–1817. doi: 10.4269/ajtmh.17-0368 29016344 PMC5805060

[11] Wiegand RE, Mwinzi PNM, Montgomery SP, Chan YL, Andiego K, Omedo M, et al. A Persistent Hotspot of Schistosoma mansoni Infection in a Five-Year Randomized Trial of Praziquantel Preventative Chemotherapy Strategies. J Infect Dis. 2017 Dec 12;216(11):1425–1433. doi: 10.1093/infdis/jix496 28968877 PMC5913648

[12] Koukounari A, Gabrielli AF, Toure S, Bosque-Oliva E, Zhang Y, et al. 2007. *Schistosoma haematobium* infection and morbidity before and after large-scale administration of praziquantel in Burkina Faso. J Infect Dis 196: 659–669. doi: 10.1086/520515 17674306

[13] Satayathum SA, Muchiri EM, Ouma JH, Whalen CC, King CH. 2006. Factors affecting infection or reinfection with *Schistosoma haematobium* in coastal Kenya: survival analysis during a nine-year, school-based treatment program. Am J Trop Med Hyg 75: 83–92. 16837713 PMC3419477

[14] Elmorshedy H, Bergquist R, El-Ela NE, Eassa SM, Elsakka EE, Barakat R. Can human Schistosomiasis mansoni control be sustained in high-risk transmission foci in Egypt? Parasit Vectors 2015; 8:372. doi: 10.1186/s13071-015-0983-2 26174621 PMC4502643

[15] Assare RK, Tian-Bi YN, Yao PK, et al. Sustaining control of Schistosomiasis mansoni in Western Cote d’Ivoire: results from a SCORE Study, one year after initial praziquantel administration. PLoS Negl Trop Dis 2016; 10:e0004329. doi: 10.1371/journal.pntd.0004329 26789749 PMC4720284

[16] Ross AG, Olveda RM, Chy D, et al. Can mass drug administration lead to the sustainable control of schistosomiasis? J Infect Dis 2015; 211:283–9. doi: 10.1093/infdis/jiu416 25070942

[17] Tallo VL, Carabin H, Alday PP, Balolong E Jr, Olveda RM, McGarvey ST. Is mass treatment the appropriate schistosomiasis elimination strategy? Bull World Health Organ 2008;86:765–71. doi: 10.2471/blt.07.047563 18949213 PMC2649512

[18] Wang W, Wang L, Liang YS. Susceptibility or resistance of praziquantel in human schistosomiasis: a review. Parasitol Res 2012; 111:1871–7. doi: 10.1007/s00436-012-3151-z 23052781

[19] Crellen T, Walker M, Lamberton PH, et al. Reduced efficacy of praziquantel against Schistosoma mansoni is associated with multiple rounds of mass drug administration. Clin Infect Dis 2016; 63:1151–9. doi: 10.1093/cid/ciw506 27470241 PMC5064161

[20] Esrey SA, Potash JB, Roberts L, Shiff C. Effects of improved water supply and sanitation on ascariasis, diarrhoea, dracunculiasis, hookworm infection, schistosomiasis, and trachoma.Bull World Health Organ 1991; 69:609–21. 1835675 PMC2393264

[21] Liang S, Seto EY, Remais JV, et al. Environmental effects on parasitic disease transmission exemplified by schistosomiasis in western China. Proc Natl Acad Sci U S A 2007; 104:7110–5. doi: 10.1073/pnas.0701878104 17438266 PMC1852328

[22] Grimes JET, Croll D, Harrison WE, Utzinger J, Freeman MC, Templeton MR. The relationship between water, sanitation and schistosomiasis: A systematic review and meta-analysis. PLoS Negl Trop Dis. 2014;8:e3296. doi: 10.1371/journal.pntd.0003296 25474705 PMC4256273

[23] Wang L, Guo J, Wu X, Chen H, Wang T, et al. (2009) China’s new strategy to block Schistosoma japonicum transmission: experiences and impact beyond schistosomiasis. Trop Med Int Health 14: 1475–1483. doi: 10.1111/j.1365-3156.2009.02403.x 19793080

[24] King JD, Endeshaw T, Escher E, Alemtaye G, Melaku S, et al. (2013) Intestinal parasite prevalence in an area of Ethiopia after implementing the SAFE strategy, enhanced outreach services, and health extension program. PLoS Negl Trop Dis 7: e2223. doi: 10.1371/journal.pntd.0002223 23755308 PMC3675016

[25] Freeman MC, Clasen T, Brooker SJ, Akoko DO, Rheingans R (2013) The impact of a school-based hygiene, water quality and sanitation intervention on soil-transmitted helminth reinfection: a cluster-randomized trial. Am J Trop Med Hyg 89: 875–883. doi: 10.4269/ajtmh.13-0237 24019429 PMC3820330

[26] WHO 1994: Report of the WHO informal consultation on hookworm infection and anemia in girls and women.

[27] Nyati-Jokomo Z, Chimbari MJ. Risk factors for schistosomiasis transmission among school children in Gwanda district, Zimbabwe. Acta Trop. 2017 Nov;175:84–90. doi: 10.1016/j.actatropica.2017.03.033 28377221

[28] Hajissa K., Muhajir A., Eshag H. A., Alfadel A., Nahied E., Dahab R., et al. (2018). Prevalence of schistosomiasis and associated risk factors among school children in Um-Asher Area, Khartoum, Sudan. BMC research notes, 11(1), 779. doi: 10.1186/s13104-018-3871-y 30382901 PMC6211415

[29] Kloos H, Fulford ACA, Butterworth AE, Sturrock RF, Ouma JM, Kariuki HC, et al 1997. Spatial patterns of human water contact and Schistosoma mansoni transmission and infection in four rural areas in Machakos District, Kenya. Soc Sci Med 44: 949–968. doi: 10.1016/s0277-9536(96)00218-3 9089917

[30] Tayo MA, Pugh RN, Bradley AK. Malumfashi Endemic Diseases Research Project, XI. Water-contact activities in the schistosomiasis study area. Ann Trop Med Parasitol. 1980;74:347–54. doi: 10.1080/00034983.1980.11687351 7396567

[31] Black CL, Mwinzi PNM, Muok EMO, Abudho B, Fitzsimmons CM, Dunne DW, et al. Influence of exposure history on the immunology and development of resistance to human schistosomiasis mansoni. PLoS Negl Trop Dis. 2010;4:e637. doi: 10.1371/journal.pntd.0000637 20351784 PMC2843635

[32] Pinot de Moira A, Fulford AJ, Kabatereine NB, Kazibwe F, Ouma JH, Dunne DW, et al. Microgeographical and tribal variations in water contact and Schistosoma mansoni exposure within a Ugandan fishing community. Trop Med Int Health. 2007;12:724–35. doi: 10.1111/j.1365-3156.2007.01842.x 17550469

[33] Grimes JET, Croll D, Harrison WE, Utzinger J, Freeman MC, Templeton MR. The roles of water, sanitation and hygiene in reducing schistosomiasis: a review. Parasites & Vectors (2015) 8:156. doi: 10.1186/s13071-015-0766-9 25884172 PMC4377019

[34] Lima e Costa MFF, Rocha RS, Leite MLC et al. (1991) A multivariate analysis of socio-demographic factors, water contact patterns and Schistosoma mansoni infection in an endemic area in Brazil. Revista Instituto de Medicina Tropical de São Paulo, 33:58–63. 10.1590/s0036-46651991000100011 1843398

[35] Zhaowu W, Kaiming B, Liping Y, Guifeng Y, Jinhua Z, Qili L (1993) Factors contributing to reinfection with Schistosomiasis japonica after treatment in the lake region of China. Acta Tropica, 54:83–88. doi: 10.1016/0001-706x(93)90053-e 7902650

[36] Farooq M, Nielsen J, Samaan SA, Mallah MB, Allam AA. The epidemiology of Schistosoma haematobium and S. mansoni infections in the Egypt-49 project area 2. Prevalence of bilharziasis in relation to personal attributes and habits. Bull World Health Organ. 1966;35:293–318. 5297627 PMC2476086

[37] Dalton P (1976) A socio-ecological approach to the control of Schistosoma mansoni in St Lucia. Bulletin of the World Health Organization 54: 587–595. 1088407 PMC2366481

[38] Utzinger J, Raso G, Brooker S, de Savigny D, Tanner M, Ørnbjerg N, et al. Schistosomiasis and neglected tropical diseases: towards integrated and sustainable control and a word of caution. Parasitology. 2009;136:1859–74. doi: 10.1017/S0031182009991600 19906318 PMC2791839

[39] Freeman MC, Ogden S, Jacobson J, Abbott D, Addiss DG, Amnie AG, et al. Integration of water, sanitation, and hygiene for the prevention and control of neglected tropical diseases: a rationale for inter-sectoral collaboration. PLoS Negl Trop Dis. 2013;7:e2439. doi: 10.1371/journal.pntd.0002439 24086781 PMC3784463

