# Supplementary material for: Unprotected water sources and low latrine coverage are contributing factors to persistent hotspots for schistosomiasis in western Kenya
Source: PLoS One. 2021 Sep 17;16(9):e0253115. doi: 10.1371/journal.pone.0253115 (PMC8448362; doi:10.1371/journal.pone.0253115)
Supplement: S1 File — (PDF) [file pone.0253115.s002.pdf]

APPENDIX 1: SCHOOL TEACHER QUESTIONNAIRE

SCHOOL WASH SURVEY FORM
[ADMINISTER QUESTIONNAIRE TO THE TEACHER]

1. Name of School________________________
2. Location____________________________
3. Number of female students in school________________
4. Number of male students in the school_____________
5. Number of teachers in school________________
6. Designation of interviewee :_______________

7. Have any previous (or subsequent) interventions concerning sanitation, hygiene, or drinking water been implemented in your school?

8. What results do you think the [name project evaluated] intervention has brought to the school?

9. Have there been any benefits for the school and wider community?

10. Has there been any impact on the number of days children miss due to illness? (try to find quantitative statistics

Focus on hygiene

11. Are there any hand washing facilities in the school? Yes [ ] No [ ]

12. How many hand-washing facilities are available?

13. Are they accessible to small children and children with disabilities?

14. Yes, all facilities are accessible [ ] some are [ ] none [ ]

15. What is the condition of these facilities?
[ ] Functioning

[ ] Not functioning

16. Where are the facilities located?
    [ ] Next to the toilet
    [ ] Near teachers staff room
    [ ] Near students classrooms
    [ ] Other----------------------

17. Is water available?

    [ ] Yes
    [ ] No

18. Is soap available?
    [ ] Yes
    [ ] No

Focus on water

19. What is the source of water in the school for

    (a) Hand washing
    [ ] Unprotected spring
    [ ] Protected spring
    [ ] Unprotected dug well
    [ ] Protected dug well
    [ ] Hand pump/tube well/borehole
    [ ] Surface water (river, creek, dam, lake, stream, canal)
    [ ] Public piped water/tap/standpipe
    [ ] Rain water collection
    [ ] In school piped water/tap/standpipe
    [ ] Mobile water tanker
    [ ] Other (specify)_________________

20. (b) Drinking

    [ ] Unprotected spring
    [ ] Protected spring
    [ ] Unprotected dug well

[ ] Protected dug well
[ ] Hand pump/tube well/borehole
[ ] Surface water (river, creek, dam, lake, stream, canal)
[ ] Public piped water/tap/standpipe
[ ] Rain water collection
[ ] In school piped water/tap/standpipe
[ ] Mobile water tanker
[ ] Other (specify) ________________

21. How do children drink?
[ ] With their own cups
[ ] With one single cup
[ ] With their hands
[ ] With their mouths

22. Which source did you observe children using most frequently?

23. What is the approximate distance from the main drinking water source to the school?

Focus on sanitation

24. Is there a toilet in the school?

Yes [ ] No [ ]

25. If yes, ask to visit the toilet –what type of toilet is it?
[ ] Pit latrine without slab or pit
[ ] Ventilated improved pit latrine (VIP)
[ ] Flush or pour flush toilet
[ ] Other _______________________

26. Observe the number of toilets available

For girls functional [ ] partially functional not functional Number--------
For boys functional [ ] partially functional not functional Number---------

27. Is there water for washing hands after using the toilet
[ ] Always
[ ] Sometimes

[ ] Never

28. Observer: What is the condition of the toilet?

[ ] Poor (presence of flies, offensive odour and visible stool on the floor,
absence of roof/door
[ ] Fair (presence of roof/door but dirty floor, flies and odour)
[ ] Moderate (presence of roof and door, clean floor,
i. some flies and odour)
[ ] Good (Clean, odourless, no flies, presence of roof and floor) 3
[ ] Excellent (Very clean, odourless, presence of roof, door and Availability of water)

29. Is girls' toilet separate from boys toilet?
Yes [ ] No [ ]

30. Are there toilets in school designed for the younger children?

Yes [ ] No [ ]

31. Is hygiene taught at school? Yes [ ] No [ ]

32. How is hygiene taught at the school

[ ] As an integral part of a special module on healthy living/life skills
[ ] As a stand-alone special module on hygiene exclusively
[ ] Through school-sponsored extracurricular programmes (e.g. Sanitation Clubs)
[ ] Only sporadically/informally/occasionally

Focus on sanitation

33. How is solid waste (garbage, rubbish) disposed at the school? (If necessary, use observation)

[ ] Thrown on a garbage dump within or near the school grounds
[ ] Buried within or near the schools grounds
[ ] Burned within or near the school grounds
[ ] Collected and taken away by a waste disposal service
[ ] Other (specify)
[ ] Don't know

34. How often is solid waste disposed of or collected?
    [ ] At least once a day
    [ ] Between once every two days and once a week
    [ ] Less frequently than once a week
    [ ] Other (specify)
    [ ] Don't know

35. At the time of the visit, are the pits/composting chambers/septic tanks obviously too full or over-flowing?

    [ ] Yes, in all facilities visited
    [ ] Yes, in more than 50% of the facilities visited
    [ ] Yes, but only in 50% or fewer of the facilities visited
    [ ] No, in none of the facilities visited
    [ ] Unable to observe

APPENDIX 2: PUPIL QUESTIONNAIRE

WASH CHILD BEHAVIOR FORM
[ADMINSTER QUESTIONNAIRE TO PUPIL]

DATE _____/_____/_____
DISTRICT NAME ___________________________CODE____________
SCHOOL NAME_______________________________CODE___________
CHILD ID________________

1. When you are at school, where do you usually go to URINATE?
    [ ] In the school toilet
    [ ] Around the school compound
    [ ] Outside of school compound
    [ ] I wait/hold it
    [ ] Others (specify) _______________
    [ ] Don't know
    Give reasons for your choice____
    ____
    ____

2. When you are at school, where do you usually go to DEFECATE?

    [ ] In the school toilet

[ ] Around the school compound
[ ] Outside of school compound
[ ] I wait/hold it
[ ] Others (specify)________________
[ ] Don't know
Give reasons for your choice
____
______

____

3. Where do you urinate while at home?
   [ ] In the school toilet
   [ ] Around the school compound
   [ ] Outside of school compound
   [ ] Others (specify)________________
   [ ] Don't know
   Give reasons for your choice
4. Where do you defecate while at home?
   [ ] In the school toilet
   [ ] Around the school compound
   [ ] Outside of school compound
   [ ] Others (specify)________________
   [ ] Don't know
   Give reasons for your choice

5. Are there water bodies near school/home?

   Yes [ ] No [ ]
   Name them --

6. If there are water bodies, which of the following activities do you perform?
   [ ] Bathing
   [ ] Washing clothes, dishes
   [ ] Fishing
   [ ] Crossing water
   [ ] Fetching water
   [ ] Playing
   [ ] Swimming
   [ ] Other (Specify)___________________

7. How often do you go there in a week?
   [ ] Every day
   [ ] Every other day

[ ] Three times
[ ] Less than three times

8. Do you think you can get any disease for going to the water bodies often?
   Yes [ ]   No [ ]

9. If so, what diseases?
   i)
   ii)
   iii)
   iv)

APPENDIX 3: HOUSE HOLD HEAD QUESTIONNAIRE

House hold questionnaire

Demographic characteristics

1. ID code interviewee: [ ]
2. What is your gender? Male Female
3. What is your age? [ ][ ] Years
4. What is your date of birth? D D [ ][ ] / M M [ ][ ] / Y Y Y Y [ ][ ][ ][ ]
5. Level of Education

☐ None ☐ Lower Primary ☐ Upper Primary

☐ Primary level ☐ Some Secondary level ☐ Secondary level ☐ Post Secondary

Socio- economic information

6. What is your main income generating activity? **(family bread winner)**

Farmer [ ] Salaried worker (teacher, nurse, etc) [ ] own business [ ] Fishing [ ]

Skilled labour (e.g carpenter, tailor, jua kali) [ ] Unskilled labour (e.g Shamba, construction) [ ]

Other, Specify …………………………………………………………………

7. In the house where the child lives, is there

[ ] Electricity
[ ] Refrigerator
[ ] Car
[ ] Cell phone
[ ] Radio
[ ] Bicycle
[ ] Telephone- landline
[ ] Television
[ ] Motorcycle
[ ] A domestic worker

8. Who makes the decisions for the home

[ ] Father

[ ] Mother

[ ] Grandparent

[ ] Other relative

9. The bread winner (s) in the household.

[ ] Father

[ ] Mother

[ ] Grandparent

[ ] Other relative

10. Floor of family home

[ ] Dirt

[ ] Mixed

[ ] Cement

11. What is the principal roofing material in the house where your child lives?

[ ] Natural material roofing such as grass reeds
[ ] Corrugated iron
[ ] Roofing tiles
[ ] Other roofing

Knowledge on Bilharzia and treatment

12. Are you aware of any water related diseases in your community? Yes No

13.If yes, Name them ------------ ------------------- -------------------- ----------------- --------------

14. Have you heard of Bilharzia before? Yes No

15. If yes, what do you know about how people can be

(a) Infected --

(b) Treated --

----

16. Has your child ever been treated for bilharzia or any other worms? Yes No

17. If yes when was it and what treatment was he/she given ---

----

Water, Sanitation and Hygiene

18. Is there a toilet in the compound? Yes [ ] No [ ]

19. What is the principal type of toilet facility used by members of your household?
    [ ] Own flush toilet
    [ ] Shared flush toilet
    [ ] Pit latrine
    [ ] VIP latrine
    [ ] Bush, field as latrine
    [ ] Other type of latrine

20. The toilet and bathroom facilities are

    [ ] Built outside the house and children also go there

    [ ] Built outside the house but children do not use there

    [ ] Built inside the house

21. Presence of a leakey tin near the toilet

Yes [ ]   No [ ]

22. What is your main source of water for your household activities?

[ ] Tapped water inside the house

[ ] Tapped water outside the house

[ ] We buy water in the estate

[ ] Borehole in the compound

[ ] We get water from the Lake

23. What is the principle household source of drinking water?

[ ] Piped drinking water in residence
[ ] Piped drinking water in public tap
[ ] Inside well drinking water
[ ] Public well
[ ] Rain for drinking water
[ ] River, canal or surface water for drinking
[ ] Other source of drinking water

24. Do you go to the lake for your domestic or any other commercial activity?

[ ] Yes   [ ] No

25. If yes how many times do you get into contact with the lake water in a day or a week?

26. How many times does your child accompany you or other children to the lake to play or any other activity in a week?
